# Incorporation of Laboratory Test Biomarkers Into Dual Antiplatelet Therapy Score Improves Prediction of Ischemic and Bleeding Events in Post-percutaneous Coronary Intervention Patients

**DOI:** 10.3389/fcvm.2022.834975

**Published:** 2022-05-16

**Authors:** Chengming Sun, Lin Zhong, Yanqiu Wu, Chengfu Cao, Danjie Guo, Jie Liu, Lei Gong, Shouxin Zhang, Jun Sun, Yingqi Yu, Weiwei Tong, Jun Yang

**Affiliations:** ^1^Department of Clinical Laboratory, Affiliated Yantai Yuhuangding Hospital of Qingdao University, Yantai, China; ^2^Department of Cardiology, Affiliated Yantai Yuhuangding Hospital of Qingdao University, Yantai, China; ^3^Medical Information Center, Peking University People’s Hospital, Beijing, China; ^4^Department of Cardiology, Peking University People’s Hospital, Beijing, China; ^5^Biochip Laboratory, Affiliated Yantai Yuhuangding Hospital of Qingdao University, Yantai, China; ^6^Gennlife (Beijing) Technology Co., Ltd., Beijing, China

**Keywords:** percutaneous coronary intervention, dual antiplatelet therapy score, aspartate aminotransferase, red cell distribution width CV, biomarker

## Abstract

This study aimed to examine the performance of the dual antiplatelet therapy (DAPT) score in two retrospective cohorts of post-percutaneous coronary intervention (PCI) patients and to explore whether incorporating additional biomarkers could further improve the predictive power of the DAPT score. In a retrospective derivation cohort of 4,798 PCI patients, the validity of DAPT score for stratifying ischemic/bleeding risks was explored. Then, the association between the baseline status of 54 laboratory test biomarkers and ischemic/bleeding events was revealed while adjusting for the DAPT score. Combinations of individual laboratory test biomarkers that were significantly associated with ischemic/bleeding events were explored to identify the ones that improved discrimination of ischemic and bleeding events when incorporated into DAPT score. Finally, the impact of the combination of biomarkers with DAPT score was validated in an independent retrospective validation cohort of 1,916 PCI patients. Patients with a high DAPT score (DAPT score ≥ 2) had significantly higher risk of ischemic events and significantly lower risk of bleeding than patients with a low DAPT score (DAPT score < 2). Moreover, the addition of aspartate aminotransferase (AST) and red cell distribution width CV (RDW-CV) into the DAPT score further improved discrimination of ischemia and bleeding. Furthermore, the incremental predictive value of AST + RDW-CV maintained with measurements was updated at post-baseline time points. DAPT score successfully stratified the risks of ischemia/bleeding post PCI in the current cohorts. Incorporation of AST + RDW-CV into the DAPT score further improved prediction for both ischemic and bleeding events.

## Introduction

Percutaneous coronary intervention (PCI) is a non-surgical treatment procedure used for improving myocardial perfusion of the heart in patients with coronary artery disease (CAD) (1, 2). Commonly, the dual antiplatelet therapy (DAPT) is used to prevent further thrombotic complications in post-PCI patients (3, 4). However, the optimal duration of the clinical use of DAPT remains controversial as prolonged DAPT treatment is closely associated with an increased risk of bleeding (5). Thus, personalization of DAPT duration based on the prediction of a patient’s ischemic and bleeding risks is necessary (6).

DAPT score is a system that is used to assess ischemic and bleeding risks simultaneously based on an individual patient’s clinical characteristics (7). Higher score quartiles are associated with higher rates of myocardial infarction (MI) or stent thrombosis (ST) and lower rates of moderate or severe bleeding (8). It has been shown that patients with high DAPT scores (DAPT score ≥ 2) benefit from prolonged DAPT due to reduced ischemic risks and an insignificant increase in bleeding risks, while patients with low DAPT scores (DAPT score < 2) suffer from prolonged DAPT due to increased bleeding risks, without a significant reduction in MI or ST risks (9, 10). However, DAPT has not been extensively validated using more complex real-world data, since it has been developed based on clinical trial data (11). Fortunately, soluble biomarkers such as Roche Troponin T and Siemens Troponin I Ultra have revolutionized the management of acute coronary syndromes (12). These biomarkers may play a role in guiding the choice of antiplatelet agent and the optimal DAPT duration (13). A previous study has shown that platelet function testing can be used as a biomarker for the efficacy of antiplatelet drugs in DAPT (14). Although biomarkers may be useful to identify patients who are at high risk for such events, the evaluation between routinely assayed biomarkers and ischemic/bleeding risks is seriously inadequate.

In this study, based on a retrospective derivation cohort, the DAPT score was validated for stratifying ischemic and bleeding risks. Then, the association between the baseline status of 54 laboratory test biomarkers and ischemic/bleeding events was investigated while adjusting for the DAPT score. Combinations of individual laboratory test biomarkers that significantly associated with ischemic or bleeding events were explored to identify the ones that improved discrimination of ischemic and bleeding events when incorporated into the DAPT score. Finally, the prediction results were verified in an independent retrospective validation cohort. We examined the performance of DAPT score in two retrospective “real-world” all-comers cohorts, and explored whether incorporating additional biomarkers could further improve its predictive power.

## Materials and Methods

### Patients

Between June 2008 and July 2017, consecutive post-PCI patients selected from the institute’s electronic medical records (EMR) system at Yantai Yuhuangding Hospital were enrolled in the derivation cohort. Meanwhile, between October 2008 and December 2018, consecutive post-PCI patients selected from the institute’s EMR system at the Peking University People’s Hospital were enrolled in the validation cohort. Based on 2011 ACCF/AHA/SCAI guideline for PCI, all patients were recommend to receive 12 months of DAPT after surgery (15). Therefore, all enrolled PCI patients were treated uniformly for 12 months with DAPT. Patients were excluded by the following criteria: (1) missing procedure information or no stenting; (2) missing any of the DAPT score variables, including LVEF, smoking status, stent type, prior PCI, or MI information. All patients provided written informed consent for their clinical information to be used in retrospective studies. The study protocol was approved by the Ethics Committee of the Yantai Yuhuangding Hospital (Yantai, China).

### Ischemic and Bleeding Endpoints Definition

Based on the diagnosis records in the EMR system, the primary ischemic endpoint was defined as a composite of MI or ST-segment. Meanwhile, the primary bleeding endpoint was defined as the hemoglobin levels that were less than the lower limit of laboratory test records in the EMR system.

### Sustained Predictive Value of Candidate Biomarkers in Post-baseline Measurements

Unlike most DAPT score variables that are likely invariable, many of the laboratory test biomarkers may reflect the temporary condition of patients and are susceptible to changes after baseline (16). Thus, the predictive value of genuine predictors for ischemic and bleeding events should remain when measurements are updated at post-baseline time points. Thus, based on the biomarkers obtained above, the last measurements of candidate biomarkers were collected before a series of time points after baseline (every 6 months until 108 months post PCI), followed by the biomarker status update according to patients not yet experiencing an event or not censored. The added predictive values of candidate biomarkers were examined at each time point in the overall cohort.

### Statistical Analysis

Wilcoxon rank-sum test for continuous variables and Fisher exact test for categorical variables were used for the baseline characteristics comparison between the derivation cohort and the validation cohort, respectively. Cumulative incidences of ischemic and bleeding events were estimated by the Kaplan-Meier (KM) method, followed by the log-rank test. Continuous measurement of each laboratory test biomarker was transformed into categorical score based on the upper limit of the reference range (ULN) and lower limit of the reference range (LLN). Specifically, the measurement score > ULN, ≤ ULN and ≥ LLN, and < LLN was represented as 1, 0, and –1, respectively (Supplementary Table 1). Using the adjusted DAPT score as a continuous variable, the association between an individual laboratory test biomarker and ischemic/bleeding event was examined independently by Cox proportional hazards regression. Biomarkers that showed significant association with either event were selected for further analysis, and the biomarkers that were associated with both ischemic and bleeding events in the same direction [both hazard ratio (HR) > 1 or < 1] were not considered for incorporation into the DAPT score. Then, all single candidate biomarkers, as well as possible combinations between one ischemia- and one bleeding-associated biomarker were exhaustively tested for incorporation into the DAPT score. C statistic of the DAPT score (17) was used to determine whether there was an improvement in the current new model. The C index values, confidence interval (CI) values, as well as the significance levels of these two C index values for the DAPT and DAPT + labtest models were calculated using compareC package in R (18), respectively. The net reclassification improvement (NRI) and integrated discrimination improvement (IDI) were calculated to assess whether added biomarkers improved the performance of DAPT score (19). The survIDINRI in R (20) was used to calculate the value of IDI and NRI at the 30th month as time point (bootstrap: 1,000 times). To correct for multiple hypothesis testing, the Benjamini-Hochberg method was applied to revise *P*-values resulted from candidate biomarker selection and combination screening. *P* < 0.05 was considered as statistically significant. All data were analyzed using R (version: 3.5.1) software.

## Results

### Baseline Characteristics

**FIGURE 1:**
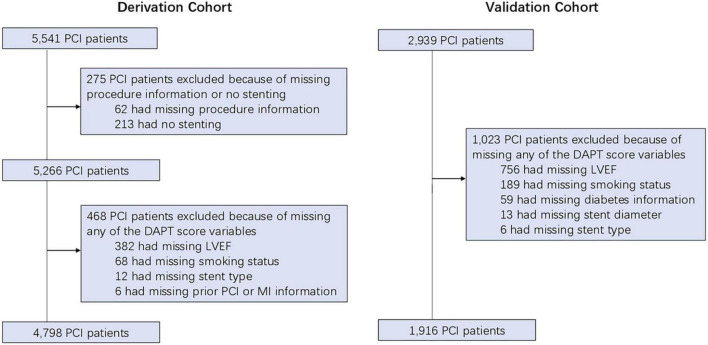
The study flow-chart. PCI, percutaneous coronary intervention; DAPT, dual antiplatelet therapy; LVEF, left ventricular ejection fraction; MI, myocardial infarction.

A total of 6,714 post-PCI patients (4,798 in the derivation cohort and 1,916 in the validation cohort) with complete data regarding DAPT score variables were included in the current study (Figure 1). The baseline information showed that the mean age of the overall cohort was 62.7 ± 10.8 years old. Among cardiovascular risk factors, hypertension was the most prevalent (63.4%), followed by smoking (32.8%) and diabetes (32.6%). The presence of a history of cardiovascular disease (prior MI 0.6%, prior stroke 7.3%) or cardiovascular intervention (prior PCI 1.5%, prior coronary artery bypass grafting 0.1%) was relatively rare. Patients predominantly received drug-eluting dents (99.9%) and clopidogrel as the P2Y_12_ inhibitor at discharge (98.3%) (Table 1). There were 881 ischemic events (18.4%) and 520 bleeding events (10.8%) during median 22.1 (10.3–37.7) months follow-up period among the 4,798 patients in derivation cohort. In validation cohort, there were 433 ischemic events (22.6%) and 251 bleeding events (13.1%) during median 30.9 (14.2–50.1) months follow-up period among the 1,916 patients.

**TABLE 1:** Baseline characteristics of PCI patients in the derivation, validation and overall cohort.

	Overall cohort (*N* = 6,714)	Derivation cohort (*N* = 4,798)	Validation cohort (*N* = 1,916)	*P*-value
**Clinical characteristics**				
Age	62.7 ± 10.8	62.4 ± 10.7	63.6 ± 11.1	<0.001
Male	4,869 (72.5%)	3,454 (72.0%)	1,415 (73.9%)	0.13
Current smoking	2,204 (32.8%)	1,586 (33.1%)	618 (32.3%)	0.55
Diabetes	2,190 (32.6%)	1,503 (31.3%)	687 (35.9%)	<0.001
Hypertension	4,256 (63.4%)	2,964 (61.8%)	1,292 (67.4%)	<0.001
Renal insufficiency	101 (1.5%)	51 (1.1%)	50 (2.6%)	<0.001
Atrial fibrillation	312 (4.6%)	215 (4.5%)	97 (5.1%)	0.30
Peripheral artery disease	8 (0.1%)	7 (0.1%)	1 (0.05%)	0.45
Prior MI	43 (0.6%)	29 (0.6%)	14 (0.7%)	0.61
Prior stroke	493 (7.3%)	325 (6.8%)	168 (8.8%)	0.005
Prior PCI	101 (1.5%)	43 (0.9%)	58 (3.0%)	<0.001
Prior CABG	10 (0.1%)	3 (0.1%)	7 (0.3%)	0.007
LVEF < 30%	38 (0.6%)	5 (0.1%)	33 (1.7%)	<0.001
Cancer	156 (2.3%)	99 (2.1%)	57 (3.0%)	0.03
**Procedure characteristics**				
Number of stents	1.62 ± 0.87	1.66 ± 0.88	1.52 ± 0.81	<0.001
Total stent length	42.1 ± 26.2	42.4 ± 25.5	41.3 ± 28.0	0.31
Minimum stent diameter	2.91 ± 0.43	2.94 ± 0.42	2.85 ± 0.45	<0.001
In-stent restenosis	65 (1.0%)	31 (0.6%)	34 (1.8%)	<0.001
Stent type				
Sirolimus-eluting stent	3,524 (52.5%)	3134(65.3%)	390(20.4%)	<0.001
Everolimus-eluting stent	1,629(24.3%)	1,005 (20.9%)	624 (32.6%)	<0.001
Zotarolimus-eluting stent	1,061 (15.8%)	334 (7.0%)	727 (37.9%)	<0.001
Paclitaxel-eluting stent	23 (0.3%)	23 (0.5%)	0 (0%)	<0.001
Bare metal stent	4 (0.06%)	1 (0.02%)	3 (0.2%)	0.07
>1 type stent	473 (7.0%)	301 (6.3%)	172 (9.0%)	<0.001
**Type of P2Y_12_ inhibitor at discharge**				
Clopidogrel	6,600 (98.3%)	4754(99.1%)	1846(96.3%)	<0.001
Ticagrelor	540 (8.0%)	540 (11.2%)	0 (0%)	<0.001

*Continuous variables were presented as mean ± standard deviation (SD). Categorical variables were presented as number (percentage). Comparisons were made between derivation and validation cohort using Wilcoxon rank-sum test for continuous variables and Fisher exact test for categorical variables. MI, myocardial infarction; CABG, coronary artery bypass grafting; LVEF, left ventricular ejection fraction.*

### Ischemic/Bleeding Risks Stratification

**FIGURE 2:**
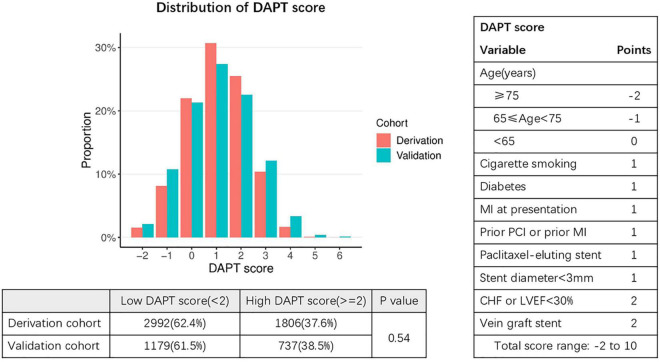
Distribution of DAPT scores in the derivation and validation cohorts. Fisher exact test was used to compare distribution of patients with low DAPT score (< 2) and high DAPT score (≥ 2) in the derivation and validation cohorts. DAPT, dual antiplatelet therapy; MI, myocardial infarction; PCI, percutaneous coronary intervention; CHF, congestive heart failure; LVEF, left ventricular ejection fraction. The X-axis represents DAPT score, while the Y-axis represents proportion.

**FIGURE 3:**
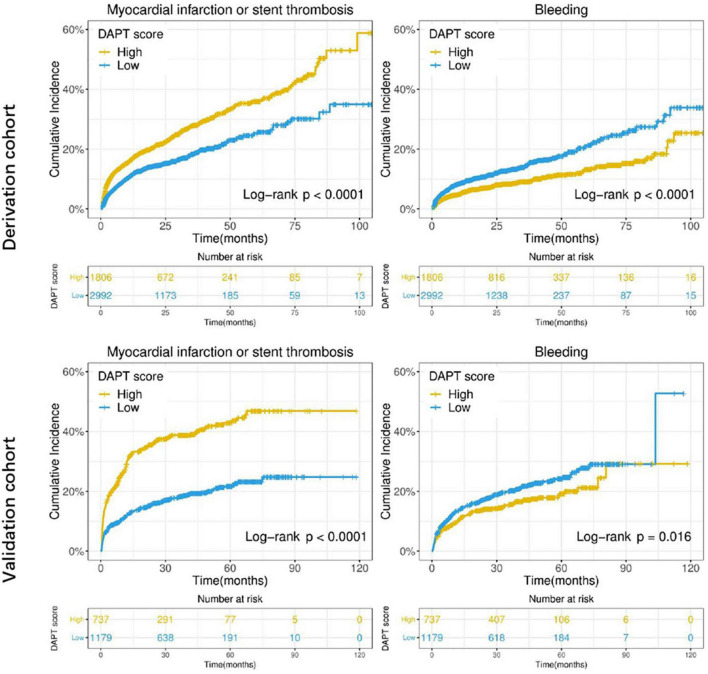
Cumulative incidence of ischemic (myocardial infarction or stent thrombosis) and bleeding events stratified by high (≥ 2) vs. low (< 2) DAPT score in the derivation and validation cohorts. The X-axis represents time (month), while the Y-axis represents cumulative cadences. *P* < 0.05 was considered to indicate statistically significant differences.

The DAPT score for the derivation cohort (-2 to 5) and the validation cohort (-2 to 6) was calculated. The results showed that the median of DAPT score was 1 in both cohorts (Figure 2). In addition, the distribution among the high DAPT group (DAPT score ≥ 2) and the low DAPT group (DAPT score < 2) were similar in both cohorts (*P* = 0.54). A significantly higher risk of ischemic events (derivation cohort: HR = 1.60; 95% CI:1.40–1.83; *P* < 0.001; validation cohort: HR = 2.45; 95% CI:2.05–2.92; *P* < 0.001) and significantly lower risk of bleeding (derivation cohort: HR = 0.61; 95% CI:0.51–0.74; *P* < 0.001; validation cohort: HR = 0.76; 95% CI:0.61–0.95; *P* = 0.016) was found in the high DAPT score group that those in the low DAPT score group (Figure 3).

### Incorporation of Laboratory Test Biomarkers Into Dual Antiplatelet Therapy Score

**FIGURE 4:**
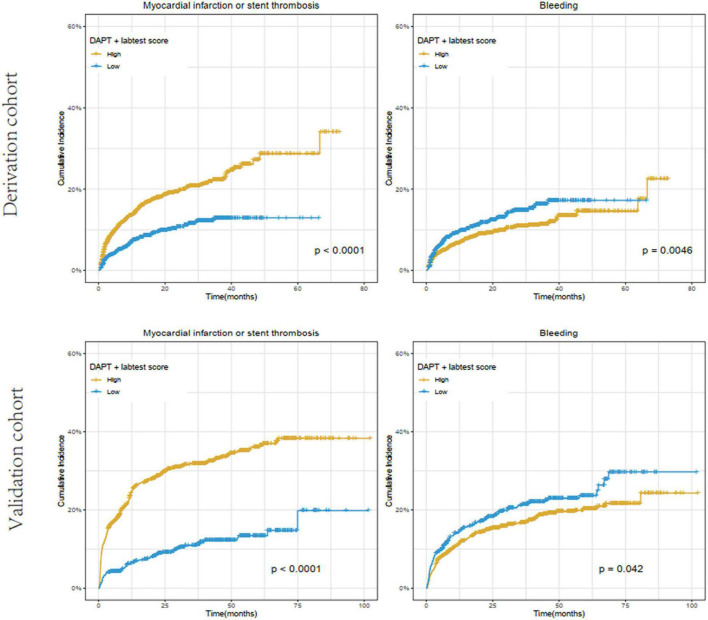
Cumulative incidence of ischemic (myocardial infarction or stent thrombosis) and bleeding events stratified by high (≥ 2) vs. low (< 2) DAPT + labtest (AST + RDW-CV) score in the derivation and validation cohorts. The X-axis represents time (month), while the Y-axis represents cumulative cadences. *P* < 0.05 was considered to indicate statistically significant differences.

Based on data availability in more than 70% of samples in the derivation cohort, a total of 54 laboratory test biomarkers were included for ischemic and bleeding events analysis (Supplementary Table 1). While adjusting for the DAPT score as a continuous variable, a total of 21 and 24 biomarkers were revealed to be significantly associated with ischemic events and bleeding events, respectively (Supplementary Table 2). After removing 14 unqualified biomarkers that were associated with both ischemic and bleeding events in the same direction (both HR > 1 or both HR < 1), a total of 7 ischemia-associated biomarkers and 10 bleeding-associated biomarkers were used as candidates for incremental predictive value investigation (Table 2). No single biomarker exhibited significant improvement in discrimination between ischemic and bleeding events when incorporated into DAPT score alone. Notably, among all possible combinations between 1 ischemia-associated biomarker and 1 bleeding-associated biomarker, the combination of aspartate aminotransferase (AST) and red cell distribution width CV (RDW-CV) showed significant improvement in discrimination between ischemia and bleeding (both *P* < 0.05) (Table 3). Similarly, high DAPT + labtest (AST + RDW-CV) score group showed higher risk of ischemic events (derivation cohort: *P* < 0.001; validation cohort: *P* < 0.0001) and lower risk of bleeding (derivation cohort: *P* = 0.0046; validation cohort: *P* = 0.042) than low DAPT + labtest score group (Figure 4).

**TABLE 2:** The candidate laboratory test biomarkers for incorporation into DAPT score.

Predictors of myocardial infarction or stent thrombosis	Sample size	HR (95% CI)	*P*-value
Alanine aminotransferase	4,378	1.29 (1.10–1.52)	0.004
Aspartate aminotransferase	4,369	1.28 (1.12–1.47)	0.002
Lactate dehydrogenase	4,365	1.59 (1.35–1.86)	<0.001
Monocyte percentage	3,631	1.37 (1.10–1.71)	0.013
Absolute monocyte count	3,631	1.38 (1.16–1.64)	0.002
Creatine kinase	4,365	1.39 (1.21–1.61)	<0.001
Total protein	4,368	0.78 (0.67–0.91)	0.003
**Predictors of bleeding**			
Alanine aminotransferase	4378	0.74 (0.58–0.93)	0.029
Aspartate aminotransferase	4,369	0.73 (0.61–0.87)	0.002
Red cell distribution width SD	3,630	1.77 (1.43–2.19)	<0.001
Red cell distribution width CV	3,630	3.39 (2.33–4.92)	<0.001
Mean corpuscular hemoglobin concentration	3,631	0.52 (0.36–0.74)	0.001
Mean corpuscular hemoglobin	3,631	0.43 (0.28–0.66)	<0.001
Low density lipoprotein cholesterol	4,366	0.72 (0.60–0.87)	0.002
Sialic acid	4,363	1.35 (1.07–1.70)	0.027
Potassium	4,383	1.65 (1.09–2.49)	0.040
Chlorine	4,382	0.67 (0.51–0.87)	0.009

*Cox proportional hazards regression was used to examine the association between individual laboratory test biomarker and ischemic and bleeding events while adjusting for DAPT score as a continuous variable. Reported P-values were corrected using Benjamini-Hochberg method to account for multiple hypothesis testing. P < 0.05 was considered to be significant different. HR, hazard ratio; CI, confidence interval; SD, standard deviation; CV, coefficient of variation.*

**TABLE 3:** The laboratory test biomarker combinations that significantly improved discrimination for both ischemic and bleeding risks when incorporated into DAPT score.

Biomarker combination				*N*	Predicted events	C (old)	C (new)	*P*	NRI	*P*	IDI	*P*
Ischemia-associated		Bleeding-associated										
biomarker		biomarker										
Name	Value	Name	Value									
AST	1 (> ULN) 0 (≤ ULN and ≥ LLN) -1 (< LLN)	RDW-CV	1 (> ULN) 0 (≤ ULN and ≥ LLN) -1 (< LLN)	3,558	Ischemia	0.590 (0.560–0.610)	0.600 (0.580–0.620)	0.040	0.118 (0.028–0.175)	0.016	0.005 (0–0.011)	0.048
					Bleeding	0.560 (0.530–0.590)	0.580 (0.550–0.610)	0.030	0.090 (0.004–0.150)	0.032	0.004 (0.001–0.009)	<0.001

*Reported P-values were corrected using Benjamini-Hochberg method to account for multiple hypothesis testing. N, sample size; C (old), C statistic of DAPT score; C (new), C statistic of DAPT score plus incorporated biomarker combination. NRI, net reclassification improvement; IDI, integrated discrimination improvement; ULN, upper limit of reference range; LLN, lower limit of reference range; AST, aspartate aminotransferase; RDW-CV, red cell distribution width coefficient of variation.*

In addition, the incorporation of AST and RDW-CV into DAPT score significantly improved the overall performance (ischemia: C statistic 0.59–0.60, *P* = 0.04; bleeding: C statistic 0.56–0.58, *P* = 0.03), as determined by the larger increase in C statistic and the smaller *P*-value in bleeding prediction. The effect of AST + RDW-CV was also confirmed by NRI (ischemia: 0.118, *P* = 0.016; bleeding: 0.092, *P* = 0.032) and IDI (ischemia: 0.005, *P* = 0.048; bleeding: 0.004, *P* < 0.001). Meanwhile, AST + RDW-CV also improved discrimination between ischemic and bleeding events (ischemia: C statistic 0.65–0.68, *P* < 0.001; bleeding: C statistic 0.54–0.56, *P* = 0.007) in the validation cohort, and the added predictive value was confirmed by NRI (ischemia: 0.28, *P* < 0.001; bleeding: 0.10, *P* = 0.022) and IDI (ischemia: 0.03, *P* < 0.001; bleeding: 0.005, *P* = 0.002).

### Sustained Predictive Value of Aminotransferase + Red Cell Distribution Width CV in the Post-baseline Measurements

To reveal the sustained predictive effect of AST + RDW-CV, the added predictive value of AST + RDW-CV was examined at each time point (every 6 months until 108 months post PCI) in the overall cohort. The results showed that the prediction value of both ischemic and bleeding events was consistently improved at all time points based on C statistic, NRI and IDI analysis (Supplementary Table 3).

## Discussion

Although DAPT score can be used to guide DAPT duration for patients undergoing PCI, the impact of ischemia/bleeding risks-related biomarkers on the predictive power of DAPT score is still unclear. In this study, DAPT score successfully stratified post-PCI ischemic and bleeding risks in two independent retrospective derivation and validation cohorts. Moreover, the minimal combination of the laboratory test biomarkers AST plus RDW-CV was revealed to improve prediction for both ischemic and bleeding events when incorporated into DAPT score. Meanwhile, the added predictive value of AST + RDW-CV persisted in the updated measurements taken every 6 months until 108 months post PCI.

Although DAPT can reduce the occurrence of ischemic events after PCI, the bleeding risk is relative high in some circumstances (21). A previous study has shown that the PRECISE-DAPT score after complex PCI was valuable in deciding DAPT duration (22). Alba and Guyatt have shown that the PRECISE-DAPT score can be successfully used to predict moderate bleeding in patients receiving DAPT after coronary stents (23). Another study has shown that the DAPT score can stratify post-PCI ischemic/bleeding events after PCI (24). A meta-analysis has suggested that the DAPT score contributes to either stratifying the risk of ischemia/bleeding or determining the best appropriate DAPT duration in patients with PCI (25). Recent guidelines for DAPT duration showed that patients with ACS should be treated for at least 12 months, unless bleeding exceeded the risk of ischemia (26). It had been suggested that longer DAPT duration could elevate the bleeding events in high-risk patients, but had no effect in the low-risk group, and displayed an obvious ischemic benefit just for low-risk group (27). Hahn et al. showed that 6-month DAPT showed more frequent myocardial infarction and wide non-inferiority margin compared with 12-month or longer DAPT, indicating prolonged DAPT should remain the standard of care in patients with acute coronary syndromes who was not at risk of excessive bleeding (28). In this study, a significant proportion of patients had follow-up information for longer than 30 months post PCI (43% of the overall cohort), which enabled us to evaluate the validity of the DAPT score in revealing the longer term ischemia/bleeding risks. In both, the derivation and validation cohort, the results showed that patients with a high DAPT score had significantly higher risk for ischemic events and significantly lower risk for bleeding compared to patients with a low DAPT score. Thus, we hypothesized that the DAPT score might be used to stratify post-PCI ischemic and bleeding risks.

AST is an important enzyme in amino acid metabolism *in vivo* (29). The levels of AST are commonly assayed in the clinic as an important biological marker for health (30). A previous study has shown that compared with patients without ischemic stroke, those with ischemic stroke have significantly higher AST (31). AST is one of the enzymes that metabolize glutamate in peripheral blood. Glutamate is the most abundant excitatory neurotransmitter and under pathological conditions as a potent neurotoxin. During the occurrence of ischemic stroke, a large amount of glutamate is produced in ischemic brain tissue, which aggravates cerebral ischemia injury and causes neurological impairment (32). Elevated AST can accelerate the metabolism of glutamate in peripheral blood, reduce the concentration of glutamate in peripheral blood, and thus promote the transport of glutamate in brain to peripheral blood along the concentration gradient (33). In the current study, the association between baseline status of 54 laboratory test biomarkers and ischemic and bleeding events were systematically examined while adjusting for the DAPT score. The results showed that AST was associated with both ischemia and bleeding risks in opposite direction. Importantly, a previous study has shown that the risk factors for ischemic and bleeding events largely overlap (34). Thus, AST might be valuable for the application of DAPT score in post-PCI. Actually, high AST levels have been shown to be associated with increased incidence of mortality in the STEMI patients after PCI (35, 36). Our results suggested that this might be attributed to increased risks of MI or ST post-PCI. Both, high monocyte percentage and monocyte count have been shown to indicate increased ischemia risk, which can be partially explained by the reported association of high monocyte count with angiographic no-reflow and high thrombus burden (37, 38).

RDW is a measure of the range of variation of red blood cell volume (39). RDW has been associated with high mortality in patients with cardiovascular disease, and has been identified as a predictor for bleeding after PCI (40, 41). A previous study has shown that there is a close relationship between RDW-CV and myocardial reperfusion after PCI in patients with CAD (42). Clinical RDW is an indicator of red blood cell heterogeneity. Increased heterogeneity of red blood cells is accompanied by decreased deformability of red blood cells, which leads to increased viscosity of red blood cells, thus slowing down blood flow velocity in microcirculation and promoting tissue ischemia and thrombosis (43). In addition, in the case of increased heterogeneity of red blood cells, the accumulation of red blood cells in the blood stream will aggravate atherosclerosis, and it is easier to form lipid accumulation and cause vasoconstriction (43, 44). On the other hand, patients with cardiovascular diseases are more likely to be complicated with oxidative stress, inflammation, malnutrition, and other conditions, which may aggravate red blood cells formation disorder, causing more obvious heterogeneity of red blood cells volume (45). These may explain the predictive value of RDW. Our results confirmed this finding by showing that high RDW-CV was associated with increased bleeding risk. Anemia has been linked to increased hemorrhagic complications and mortality following PCI (46). Our data indicated that MCH and MCHC inversely correlated with bleeding incidence.

Optimal biomarkers that improve ischemia/bleeding risks prediction when incorporated into DAPT score may be an innovation for DAPT implementation (47, 48). A previous DAPT study has shown a moderate discrimination for ischemia (C statistic 0.70) and bleeding (C statistic 0.68), since both metrics were calculated based on separate models that contained predictors for both ischemia and bleeding risks (7). As these predictors were not incorporated in the DAPT score, the discrimination power of the DAPT score was likely diminished. In this study, only marginal discrimination between ischemia (C statistic 0.58) and bleeding (C statistic 0.57) was observed with the DAPT score in our derivation cohort. Regarding the laboratory test biomarkers, AST + RDW-CV performed the best among all possible combinations of one ischemia-associated and one bleeding-associated biomarker. More importantly, based on the notion that the updated biomarker status during follow-up before adverse event occurrence should more closely reflect patient condition. The current study investigated whether post-baseline measurements of AST + RDW-CV continued to improve prediction for ischemia and bleeding risks. The prediction for both ischemic and bleeding events was consistently improved using the latest measurements collected at all post-baseline time points, which further consolidated AST + RDW-CV as a robust predictor for ischemia and bleeding risks. However, the current study had several limitations: (i) status of anti-platelet medication during the study period was not identified (for example, some patients might be continue DAPT therapy or aspirin/P2Y12 alone after the 12 months DAPT therapy), which prevented us from comparing benefit and harm due to different DAPT durations in subgroups stratified by DAPT score; (ii) the definition of bleeding endpoint in the current study differed from established bleeding criteria such as GUSTO, TIMI and BARC (49), and (iii) continuous measurements of laboratory test biomarkers were categorized according to the upper and lower limit of the reference range to facilitate model simplicity and ease of clinical adoption, which may have limited the sensitivity of detection of certain biomarkers.

## Conclusion

In conclusion, DAPT score might be valuable to stratify ischemic/bleeding risks in post-PCI patients. Moreover, AST + RDW-CV might improve prediction for both ischemic/bleeding events when incorporated into DAPT score.

## Data Availability Statement

The original contributions presented in the study are included in the article/Supplementary Material, further inquiries can be directed to the corresponding author/s.

## Ethics Statement

The studies involving human participants were reviewed and approved by the Ethics Committee of the Yantai Yuhuangding Hospital. The patients/participants provided their written informed consent to participate in this study.

## Author Contributions

JY, WT, CS, LZ, YW, and CC: conception and design of the research. JL, LG, SZ, and JS: acquisition of data. DG, JL, LG, SZ, JS, and YY: analysis, interpretation of data, and statistical analysis. CS, LZ, YW, and CC: drafting the manuscript. JY and WT: revision of manuscript for important intellectual content. All authors read and approved the final manuscript.

## Conflict of Interest

YY and WT were employed by the company Gennlife (Beijing) Technology Co., Ltd. The remaining authors declare that the research was conducted in the absence of any commercial or financial relationships that could be construed as a potential conflict of interest.

## Publisher’s Note

All claims expressed in this article are solely those of the authors and do not necessarily represent those of their affiliated organizations, or those of the publisher, the editors and the reviewers. Any product that may be evaluated in this article, or claim that may be made by its manufacturer, is not guaranteed or endorsed by the publisher.
