# Supplementary material for: Incorporation of Laboratory Test Biomarkers Into Dual Antiplatelet Therapy Score Improves Prediction of Ischemic and Bleeding Events in Post-percutaneous Coronary Intervention Patients
Source: Front Cardiovasc Med. 2022 May 16;9:834975. doi: 10.3389/fcvm.2022.834975 (PMC9148992; doi:10.3389/fcvm.2022.834975)
Supplement: Supplementary file 1 [file Data_Sheet_1.docx]

**Supplementary Table 1.** Reference range of laboratory test biomarkers examined in the derivation cohort

| **Biomarker** | **Unit** | **Reference range**  **(LLN, ULN) : N** |
| --- | --- | --- |
| **Complete blood count** | | |
| White blood cell count | x10^9/L | (3.5,9.5):3535;(4,10):93 |
| Red blood cell count | x10^12/L | (4.3,5.8):2508;(3.8,5.1):1026;(4.09,5.74):39;  (4.0,5.5):28;(3.68,5.13):16;(4.5):10;(4.0,4.5):1 |
| Platelet count | x10^9/L | (125,350):3537;(100,300):94 |
| Hemoglobin | g/L | (130,175):2510;(115,150):1026;(131.172):40;  (110,170):38;(113,151):16;(120,140):1 |
| Hematocrit | % | (40,50):2498;(35,45):1021;(35,54):95 |
| Mean corpuscular volume | fL | (82,100):3537;(80,98):94 |
| Mean corpuscular hemoglobin | pg | (27,34):3537;(27,35):94 |
| Mean corpuscular hemoglobin concentration | g/L | (316,354):3537;(320,360):94 |
| Red cell distribution width SD | fL | (39,46):3631 |
| Red cell distribution width CV | % | (10.9.14.5):3631 |
| Mean platelet volume | fL | (7.6,13.2):3631 |
| Platelet distribution width | fL | (9,17):3631 |
| Plateletcrit | % | (0.11,0.28):3631 |
| Platelet large cell ratio | % | (13,43):3631 |
| Absolute neutrophil count | x10^9/L | (1.8,6.3):3537;(2,7):94 |
| Neutrophil percentage | % | (40,75):3537;(50,70):94 |
| Absolute lymphocyte count | x10^9/L | (1.1,3.2):3537;(0.8,4.0):94 |
| Lymphocyte percentage | % | (20,50):3537;(20,40):94 |
| Absolute monocyte count | x10^9/L | (0.1,0.6):3537;(0.12,0.8):94 |
| Monocyte percentage | % | (3,10):3537;(3,9):94 |
| Absolute basophil count | x10^9/L | (0,0.06):3537;(0,0.1):94 |
| Basophil percentage | % | (0,1):3631 |
| Absolute eosinophil count | x10^9/L | (0.02,0.52):3537;(0.05,0.5):94 |
| Eosinophil percentage | % | (0.4,8):3537;(0.5,5):94 |
| **Blood chemistry** | | |
| Glucose | mmol/L | (3.8,6.2):4379 |
| Calcium | mmol/L | (2.1,2.8):2602;(2,11,2,52):1779 |
| Sodium | mmol/L | (137,147):3940;(135,145):442 |
| Potassium | mmol/L | (3.5,5.3):3940;(3.5,5.6):443 |
| Chloride | mmol/L | (99,110):3940;(96,110):442 |
| Magnesium | mmol/L | (0.67,1.10):2602;(0.75,1.02):1770 |
| Bicarbonate | mmol/L | (21,30):4374 |
| Creatinine | umol/L | (45,124):2603;(57,111):782;(45,124):687;  (57,97):493;(41,81):396;(41,73):104 |
| Creatine kinase | U/L | (22,160):2602;(50,310):1266;(40,200):497 |
| Alanine aminotransferase | U/L | (9.50):2786;(7,40):1152;(5,40):440 |
| Aspartate aminotransferase | U/L | (15,40):2781;(13,35):1148;(5,40):440 |
| Lactate dehydrogenase | U/L | (90,205):2602;(120,250):1763 |
| Gamma glutamyl transferase | U/L | (10.60):2778;(7,45):1148;(6,64):442 |
| Alkaline phosphatase | U/L | (45,125):2778;(50,135):1113;(45,125):1006;  (34,135):442;(35,100):35 |
| Total cholesterol | mmol/L | (3.12,5.72):4366 |
| High density lipoprotein cholesterol | mmol/L | (1.04,1.96):4366 |
| Low density lipoprotein cholesterol | mmol/L | (1.53,3.45):4366 |
| Lipoprotein(a) | mg/L | (0,300):4366 |
| Triglyceride | mmol/L | (0.4,1.7):4366 |
| Total protein | g/L | (65,85):3926;(60,82):442 |
| Albumin | g/L | (40,55):3926;(35,55):442 |
| Globulin | g/L | (20,40):3926;(20,35):443 |
| Albumin to globulin ratio | NA | (1.2,2.4):3926;(1.2,2.5):443 |
| Total bilirubin | umol/L | (3.2,23.5):4368 |
| Direct bilirubin | umol/L | (0,8):2605;(0,6.8):1763 |
| Indirect bilirubin | umol/L | (3,19):4369 |
| Total bile acid | umol/L | (0,14):4368 |
| Uric acid | umol/L | (208,428):3118;(149,368):1251 |
| Sialic acid | mg/L | (470,700):2600;(456,754):1763 |
| Urea | mmol/L | (2.1,7.5):2602;(3.6,9.5):782;(3.1.8.0):493;  (3.1,8.8):396;(2.6,7.5):105 |
| Inorganic phosphorus | mmol/L | (0.8,1.48):2602;(0.85,1.52):1770 |
| Anion gap | mmol/L | (8,16):3930 |

**Notes:** ULN, upper limit of reference range; LLN, lower limit of reference range; N, sample size; SD, standard deviation; CV, coefficient of variation.

**Supplementary Table 2.** Association of examined laboratory test biomarkers with ischemic and bleeding events post PCI in the derivation cohort

| **Biomarker** | **N** | **Ischemia** | | **Bleeding** | |
| --- | --- | --- | --- | --- | --- |
|  |  | **HR (95% CI)** | **P** | **HR (95% CI)** | **P** |
| **Complete blood count** | | | | | |
| White blood cell count | 3628 | 1.04(0.84-1.30) | 0.818 | 0.88(0.66-1.17) | 0.471 |
| Red blood cell count | 3628 | 0.62(0.51-0.74) | <0.001 | 0.29(0.24-0.36) | <0.001 |
| Platelet count | 3630 | 1.15(0.83-1.60) | 0.548 | 1.02(0.67-1.56) | 0.936 |
| Hemoglobin | 3631 | 0.59(0.49-0.73) | <0.001 | 0.23(0.19-0.29) | <0.001 |
| Hematocrit | 3614 | 0.62(0.52-0.73) | <0.001 | 0.25(0.20-0.30) | <0.001 |
| Mean corpuscular volume | 3631 | 1.01(0.66-1.57) | 0.948 | 0.74(0.44-1.24) | 0.362 |
| Mean corpuscular hemoglobin | 3631 | 0.80(0.54-1.18) | 0.397 | 0.43(0.28-0.66) | <0.001 |
| Mean corpuscular hemoglobin concentration | 3631 | 1.04(0.79-1.39) | 0.876 | 0.52(0.36-0.74) | 0.001 |
| Red cell distribution width SD | 3630 | 1.17(0.99-1.39) | 0.152 | 1.77(1.43-2.19) | <0.001 |
| Red cell distribution width CV | 3630 | 1.06(0.64-1.75) | 0.906 | 3.39(2.33-4.92) | <0.001 |
| Mean platelet volume | 3626 | 0.46(0.11-1.96) | 0.433 | 0.33(0.05-2.03) | 0.338 |
| Platelet distribution width | 3626 | 0.97(0.61-1.52) | 0.911 | 0.93(0.54-1.60) | 0.835 |
| Plateletcrit | 3626 | 1.46(1.18-1.80) | 0.002 | 1.39(1.07-1.81) | 0.034 |
| Platelet large cell ratio | 3626 | 1.55(1.00-2.38) | 0.118 | 1.46(0.86-2.50) | 0.261 |
| Absolute neutrophil count | 3631 | 1.12(0.91-1.37) | 0.425 | 0.90(0.69-1.18) | 0.538 |
| Neutrophil percentage | 3631 | 1.05(0.84-1.32) | 0.780 | 1.33(1.02-1.72) | 0.071 |
| Absolute lymphocyte count | 3631 | 0.67(0.54-0.83) | 0.002 | 0.53(0.41-0.68) | <0.001 |
| Lymphocyte percentage | 3631 | 0.73(0.61-0.87) | 0.002 | 0.71(0.57-0.88) | 0.006 |
| Absolute monocyte count | 3631 | 1.38(1.16-1.64) | 0.002 | 1.15(0.92-1.43) | 0.326 |
| Monocyte percentage | 3631 | 1.37(1.10-1.71) | 0.013 | 1.35(1.03-1.76) | 0.062 |
| Absolute basophil count | 3631 | 1.30(0.95-1.77) | 0.195 | 0.77(0.48-1.23) | 0.367 |
| Basophil percentage | 3631 | 0.96(0.56-1.62) | 0.911 | 1.01(0.54-1.88) | 0.984 |
| Absolute eosinophil count | 3631 | 1.23(0.90-1.68) | 0.328 | 1.12(0.78-1.61) | 0.596 |
| Eosinophil percentage | 3631 | 1.15(0.91-1.47) | 0.397 | 1.21(0.90-1.63) | 0.326 |
| **Blood chemistry** | | | | | |
| Glucose | 4379 | 1.07(0.93-1.24) | 0.485 | 1.19(0.99-1.43) | 0.140 |
| Calcium | 4381 | 0.73(0.60-0.88) | 0.004 | 0.61(0.49-0.77) | <0.001 |
| Sodium | 4382 | 0.75(0.63-0.88) | 0.002 | 0.63(0.51-0.78) | <0.001 |
| Potassium | 4383 | 0.97(0.74-1.27) | 0.906 | 1.65(1.09-2.49) | 0.040 |
| Chloride | 4382 | 0.77(0.62-0.96) | 0.054 | 0.67(0.51-0.87) | 0.009 |
| Magnesium | 4372 | 1.09(0.79-1.51) | 0.735 | 0.82(0.52-1.29) | 0.474 |
| Bicarbonate | 4375 | 0.98(0.81-1.19) | 0.911 | 0.84(0.66-1.06) | 0.233 |
| Creatinine | 4378 | 1.53(1.19-1.96) | 0.003 | 3.37(2.54-4.46) | <0.001 |
| Creatine kinase | 4365 | 1.39(1.21-1.61) | <0.001 | 0.92(0.77-1.11) | 0.471 |
| Alanine aminotransferase | 4378 | 1.29(1.10-1.52) | 0.004 | 0.74(0.58-0.93) | 0.029 |
| Aspartate aminotransferase | 4369 | 1.28(1.12-1.47) | 0.002 | 0.73(0.61-0.87) | 0.002 |
| Lactate dehydrogenase | 4365 | 1.58(1.35-1.86) | <0.001 | 0.86(0.71-1.04) | 0.197 |
| Gamma glutamyl transferase | 4368 | 1.07(0.86-1.33) | 0.709 | 0.92(0.68-1.25) | 0.634 |
| Alkaline phosphatase | 4368 | 0.76(0.56-1.02) | 0.152 | 0.88(0.61-1.29) | 0.586 |
| Total cholesterol | 4366 | 0.88(0.76-1.02) | 0.179 | 0.84(0.70-1.02) | 0.147 |
| High density lipoprotein cholesterol | 4366 | 0.77(0.66-0.89) | 0.002 | 0.76(0.63-0.91) | 0.009 |
| Low density lipoprotein cholesterol | 4366 | 1.04(0.90-1.20) | 0.735 | 0.72(0.60-0.87) | 0.002 |
| Lipoprotein(a) | 4366 | 1.10(0.94-1.28) | 0.397 | 1.02(0.83-1.24) | 0.892 |
| Triglyceride | 4366 | 0.96(0.82-1.12) | 0.735 | 0.92(0.75-1.13) | 0.521 |
| Total protein | 4368 | 0.78(0.67-0.90) | 0.003 | 0.85(0.70-1.03) | 0.169 |
| Albumin | 4368 | 0.72(0.62-0.85) | 0.001 | 0.64(0.51-0.79) | <0.001 |
| Globulin | 4369 | 1.01(0.74-1.38) | 0.948 | 1.39(0.88-2.18) | 0.258 |
| Albumin to globulin ratio | 4369 | 0.72(0.59-0.86) | 0.002 | 0.55(0.45-0.68) | <0.001 |
| Total bilirubin | 4368 | 1.19(0.95-1.47) | 0.237 | 0.86(0.62-1.18) | 0.450 |
| Direct bilirubin | 4368 | 1.21(0.63-2.33) | 0.735 | 1.93(0.96-3.89) | 0.135 |
| Indirect bilirubin | 4369 | 1.22(0.99-1.51) | 0.146 | 0.91(0.67-1.22) | 0.586 |
| Total bile acid | 4368 | 0.62(0.29-1.30) | 0.350 | 1.68(0.94-2.97) | 0.147 |
| Uric acid | 4369 | 1.33(1.13-1.57) | 0.003 | 1.45(1.18-1.77) | 0.001 |
| Sialic acid | 4363 | 1.18(0.98-1.42) | 0.160 | 1.35(1.07-1.70) | 0.027 |
| Urea | 4378 | 1.40(1.14-1.72) | 0.004 | 2.11(1.68-2.65) | <0.001 |
| Inorganic phosphorus | 4372 | 0.91(0.72-1.15) | 0.568 | 1.16(0.85-1.59) | 0.450 |
| Anion gap | 3930 | 1.27(0.93-1.74) | 0.239 | 0.77(0.49-1.22) | 0.367 |

**Notes:** Reported P values were corrected using Benjamini-Hochberg method to account for multiple hypothesis testing. N, sample size; HR, hazard ratio; CI, confidence interval; SD, standard deviation; CV, coefficient of variation.

**Supplementary Table 3.** Improvement in prediction for ischemic and bleeding risks when AST plus RDW-CV were incorporated into DAPT score using last measurements by post-baseline time points in the overall cohort

| **Time point (month)** | **Predicted events** | **C (old)** | **C (new)** | **P** | **NRI** | **P** | **IDI** | **P** |
| --- | --- | --- | --- | --- | --- | --- | --- | --- |
| 0 | Ischemia | 0.585(0.561~0.61) | 0.599(0.575~0.623) | 0.013 | 0.187(0.121~0.226) | <0.001 | 0.012(0.006~0.018) | <0.001 |
|  | Bleeding | 0.557(0.526~0.587) | 0.579(0.548~0.61) | 0.002 | 0.094(0.044~0.141) | 0.002 | 0.005(0.002~0.009) | <0.001 |
| 6 | Ischemia | 0.585(0.561~0.61) | 0.601(0.577~0.625) | 0.004 | 0.166(0.092~0.203) | 0.002 | 0.011(0.005~0.017) | <0.001 |
|  | Bleeding | 0.557(0.526~0.587) | 0.577(0.546~0.608) | 0.005 | 0.086(0.041~0.128) | 0.006 | 0.004(0.002~0.007) | <0.001 |
| 12 | Ischemia | 0.585(0.561~0.61) | 0.602(0.578~0.626) | 0.002 | 0.168(0.113~0.206) | <0.001 | 0.011(0.005~0.017) | <0.001 |
|  | Bleeding | 0.557(0.526~0.587) | 0.576(0.546~0.607) | 0.005 | 0.077(0.033~0.121) | 0.01 | 0.004(0.002~0.007) | <0.001 |
| 18 | Ischemia | 0.585(0.561~0.61) | 0.602(0.578~0.627) | 0.002 | 0.169(0.128~0.207) | <0.001 | 0.012(0.006~0.018) | <0.001 |
|  | Bleeding | 0.557(0.526~0.587) | 0.575(0.544~0.606) | 0.008 | 0.068(0.02~0.111) | 0.026 | 0.004(0.001~0.006) | <0.001 |
| 24 | Ischemia | 0.585(0.561~0.61) | 0.603(0.578~0.627) | 0.001 | 0.17(0.124~0.209) | <0.001 | 0.011(0.005~0.017) | <0.001 |
|  | Bleeding | 0.557(0.526~0.587) | 0.575(0.544~0.606) | 0.009 | 0.071(0.026~0.114) | 0.02 | 0.004(0.002~0.007) | <0.001 |
| 30 | Ischemia | 0.585(0.561~0.61) | 0.603(0.578~0.627) | 0.001 | 0.17(0.125~0.209) | <0.001 | 0.011(0.005~0.017) | <0.001 |
|  | Bleeding | 0.557(0.526~0.587) | 0.575(0.544~0.606) | 0.009 | 0.068(0.022~0.111) | 0.022 | 0.004(0.002~0.007) | <0.001 |
| 36 | Ischemia | 0.585(0.561~0.61) | 0.603(0.578~0.627) | 0.001 | 0.168(0.12~0.207) | <0.001 | 0.01(0.005~0.017) | <0.001 |
|  | Bleeding | 0.557(0.526~0.587) | 0.575(0.544~0.606) | 0.01 | 0.071(0.026~0.114) | 0.02 | 0.004(0.002~0.007) | <0.001 |
| 42 | Ischemia | 0.585(0.561~0.61) | 0.603(0.579~0.627) | 0.001 | 0.167(0.119~0.204) | <0.001 | 0.01(0.005~0.017) | <0.001 |
|  | Bleeding | 0.557(0.526~0.587) | 0.575(0.544~0.606) | 0.01 | 0.071(0.026~0.114) | 0.02 | 0.004(0.002~0.007) | <0.001 |
| 48 | Ischemia | 0.585(0.561~0.61) | 0.603(0.579~0.627) | 0.001 | 0.169(0.123~0.209) | <0.001 | 0.011(0.005~0.017) | <0.001 |
|  | Bleeding | 0.557(0.526~0.587) | 0.575(0.544~0.606) | 0.01 | 0.07(0.026~0.112) | 0.02 | 0.004(0.002~0.007) | <0.001 |
| 54 | Ischemia | 0.585(0.561~0.61) | 0.603(0.579~0.627) | 0.001 | 0.169(0.122~0.209) | <0.001 | 0.011(0.005~0.017) | <0.001 |
|  | Bleeding | 0.557(0.526~0.587) | 0.575(0.544~0.606) | 0.01 | 0.07(0.023~0.112) | 0.022 | 0.004(0.002~0.007) | <0.001 |
| 60 | Ischemia | 0.585(0.561~0.61) | 0.603(0.579~0.627) | 0.001 | 0.169(0.122~0.209) | <0.001 | 0.01(0.005~0.017) | <0.001 |
|  | Bleeding | 0.557(0.526~0.587) | 0.575(0.544~0.606) | 0.01 | 0.071(0.024~0.113) | 0.022 | 0.004(0.002~0.007) | <0.001 |
| 66 | Ischemia | 0.585(0.561~0.61) | 0.603(0.579~0.627) | 0.001 | 0.169(0.123~0.209) | <0.001 | 0.011(0.005~0.017) | <0.001 |
|  | Bleeding | 0.557(0.526~0.587) | 0.575(0.544~0.606) | 0.01 | 0.07(0.022~0.112) | 0.026 | 0.004(0.001~0.007) | <0.001 |
| 72 | Ischemia | 0.585(0.561~0.61) | 0.603(0.579~0.627) | 0.001 | 0.169(0.123~0.209) | <0.001 | 0.011(0.005~0.017) | <0.001 |
|  | Bleeding | 0.557(0.526~0.587) | 0.575(0.544~0.606) | 0.01 | 0.07(0.022~0.112) | 0.026 | 0.004(0.001~0.007) | <0.001 |
| 78 | Ischemia | 0.585(0.561~0.61) | 0.603(0.579~0.627) | 0.001 | 0.169(0.123~0.209) | <0.001 | 0.011(0.005~0.017) | <0.001 |
|  | Bleeding | 0.557(0.526~0.587) | 0.575(0.544~0.606) | 0.01 | 0.069(0.022~0.112) | 0.026 | 0.004(0.001~0.007) | <0.001 |
| 84 | Ischemia | 0.585(0.561~0.61) | 0.603(0.579~0.627) | 0.001 | 0.169(0.123~0.209) | <0.001 | 0.011(0.005~0.017) | <0.001 |
|  | Bleeding | 0.557(0.526~0.587) | 0.575(0.544~0.606) | 0.01 | 0.069(0.022~0.112) | 0.026 | 0.004(0.001~0.007) | <0.001 |
| 90 | Ischemia | 0.585(0.561~0.61) | 0.603(0.579~0.627) | 0.001 | 0.169(0.123~0.209) | <0.001 | 0.011(0.005~0.017) | <0.001 |
|  | Bleeding | 0.557(0.526~0.587) | 0.575(0.544~0.606) | 0.01 | 0.069(0.022~0.112) | 0.026 | 0.004(0.001~0.007) | <0.001 |
| 96 | Ischemia | 0.585(0.561~0.61) | 0.603(0.579~0.627) | 0.001 | 0.169(0.123~0.209) | <0.001 | 0.011(0.005~0.017) | <0.001 |
|  | Bleeding | 0.557(0.526~0.587) | 0.575(0.544~0.606) | 0.01 | 0.069(0.022~0.112) | 0.026 | 0.004(0.001~0.007) | <0.001 |
| 102 | Ischemia | 0.585(0.561~0.61) | 0.603(0.579~0.627) | 0.001 | 0.169(0.123~0.209) | <0.001 | 0.011(0.005~0.017) | <0.001 |
|  | Bleeding | 0.557(0.526~0.587) | 0.575(0.544~0.606) | 0.01 | 0.069(0.022~0.112) | 0.026 | 0.004(0.001~0.007) | <0.001 |
| 108 | Ischemia | 0.585(0.561~0.61) | 0.603(0.579~0.627) | 0.001 | 0.169(0.123~0.209) | <0.001 | 0.011(0.005~0.017) | <0.001 |

**Notes:** C(old), C statistic of DAPT score; C(new), C statistic of DAPT score plus AST and RDW-CV. NRI, net reclassification improvement; IDI, integrated discrimination improvement.
